# Pollination efficiency of hummingbirds and flowerpiercers at the flowers of *Lobelia laxiflora* (Campanulaceae): morphological fit matters

**DOI:** 10.1007/s00442-025-05718-z

**Published:** 2025-04-30

**Authors:** Stefan Abrahamczyk, Ruben Dürr, Emanuel Brenes, María A. Maglianesi

**Affiliations:** 1 Botanical Garden, University of Osnabrück, Albrechtstraße 29, 49076 Osnabrück, Germany; 2 Botany Department, State Museum of Natural History Stuttgart, Rosenstein 1, 70191 Stuttgart, Germany; 3 School of Agronomy, Costa Rica Institute of Technology (ITCR), San Carlos Campus, Alajuela, 223-21002 Costa Rica

**Keywords:** *Colibri*, *Diglossa*, Nectar robbing, Seed set, Self-incompatibility

## Abstract

Research on pollination systems has largely focused on structures of mutualistic networks, whereas pollinator efficiency defining the quality of visits received much less attention. Different flower-visiting animals can vary in their pollination efficiency, e.g. due to their morphology, size or visitation frequency. Here, we analyse several reproductive traits, including flower morphology and reproductive system of *Lobelia laxiflora* and compare pollination efficiency of flower visitors based on seed set. We found experimentally that *Lobelia laxiflora* is completely self-incompatible and that the flowers are frequently visited by *Colibri cyanotus*, which did not show preferences for one flower sex. *Diglossa plumbea* was a more rare visitor and concentrated on female flowers *Diglossa* forced their bills deeply into the dorsally open corolla tube but did not pierce flowers. Corolla tube length perfectly fitted bill length of *Colibri cyanotus* and lots of pollen was deposited on its heads. In contrast, *Diglossa plumbea* visited flowers by sitting in different positions to them. Therefore, the reproductive flower organs got in contact with different parts of its body. Consequently, *Colibri cyanotus* was a very efficient pollinator probably due to the high level of trait matching, whereas *Diglossa plumbea* was not pollinating at all. In conclusion, our study documents a rare case of a temporally limited one-to-one dependency of a plant and a hummingbird species on the population level. Additionally, it highlights the significant role of morphological trait matching and bird´s behaviour in flower handling for efficient pollination and demonstrates that non-adapted flower visitors may fail as pollinators.

## Introduction

More than 530 bird species are known to visit and pollinate flowers in the Neotropics, ranging from opportunistic, mostly passerine birds, feeding on floral resources only occasionally to highly specialized nectar feeders, such as hummingbirds (Stiles 1981; Rocca and Sazima 2010; Barreto et al. 2024). Nectar-feeding birds show a range of morphological variation in their bills and tongues as well as in their ability to digest sucrose depending on the proportion of nectar at their diet (Nicolson and Fleming 2003). This variation in the specialization degree of flower-visiting birds is reflected in floral traits of their food plants, e.g. in corolla length or nectar sugar composition (Abrahamczyk 2019). Therefore, most bird-pollinated plant species are either pollinated largely by generalist or by specialist nectar-feeding birds (Abrahamczyk 2019).

Within the group of specialized nectar-feeding birds a few species, e.g. in the genera *Diglossa*, *Schistes* or *Lesbia* are adapted to nectar robbing (Naoki 1998; Maloof and Inouye 2000; Boehm 2018; Igić et al. 2020), which means that they commonly visit long-tubed, nectar-rich flowers (Ornelas et al. 2007) and pierce them at the base of the corolla to get access to the nectar. Even though these birds are not obligate robbers, a large proportion of their diet comes from primary (active piercing) or secondary nectar robbing (using existing holes; Colwell et al. 1974; Igić et al. 2020).

From the plant´s perspective, nectar robbing is not necessarily negative as long as the reproductive parts of the flowers are not destroyed (Maloof and Inouye 2000; Heiling et al. 2018). Nectar robbing often reduces nectar quality and quantity and makes plants less attractive for territorial pollinators (Hazlehurst and Karubian 2016). Thus, flowers are more often visited by non-territorial pollinators, which use larger areas to search for food than territorial species, visit more plant individuals and, therefore, increase outcrossing (Maloof and Inouye 2000; Hazlehurst and Karubian 2016). Many hummingbird-pollinated plant species are largely self-incompatible (Abrahamczyk et al. 2022) and may thus indirectly profit from nectar robbing.

Despite the indirect effects, nectar-robbing birds, especially flowerpiercers (*Diglossa* ssp.) can also directly impact pollination. Movement-induced self-pollination by nectar-robbing birds has been observed in *Disterigma stereophyllum* (Ericaceae; Navarro et al. 2008). Further, Graves (1982) documented pollination of *Tristerix longebracteatus* (Loranthaceae) flowers by birds getting in contact with stigmas and stamens of lots of flowers per flower cluster while piercing the central flowers at the base. And finally, flowerpiercers legitimately visit the short-tubed flowers of *Calliandra grandifolia* (Fabaceae) and *Cirsium jaliscoense* (Asteraceae; Arizmendi 2001) and have also been observed at the long-tubed flowers of *Lobelia laxiflora* (Campanulaceae; Schondube and del Rio 2003).

The observations of the above-mentioned studies involving non-destructive flower visits of flowerpiercers lead to the question on the pollination efficiency of these birds in comparison to hummingbirds, the actual pollinators of the flowers. Pollination efficiency is defined as the contribution of a pollinator to female plant fitness, i.e. seed set (Ne'eman et al. 2010). Many plant species are known to have primary and secondary pollinator groups that vary in their morphological adaptation and pollination efficiency (Rosas-Guerrero et al. 2014). Pollination efficiency of different pollinator groups varies from nearly equal among groups to complete inefficiency of a single group and the most efficient group determines the pollination syndrome (Rosas-Guerrero et al. 2014). Beside pollination efficiency also, the frequency of visits is important for seed production. If a pollinator with lower pollination efficiency is much more common than a pollinator with higher pollination efficiency, it may contribute equally or even more to seed set of a given plant species (Fumero‐Cabán and Meléndez‐Ackerman 2007; Leal et al. 2020). These contributions may also vary among years.

During a field study in Costa Rica, we observed that *Diglossa plumbea* apparently legitimately visited the long-tubed flowers of *Lobelia laxiflora*. Since *Lobelia laxiflora* was also—but in much higher frequency—visited by *Colibri cyanotus*, we set up an experiment to analyse the pollination ecology as well as the reproductive system of the plant species. Specifically, we ask 1. How frequently do *Diglossa plumbea* and *Colibri cyanotus* visit the flowers of *Lobelia laxiflora*? 2. Are both flower visitor species similarly efficient pollinators? 3. Does selfing play a role in the pollination of *Lobelia laxiflora*?

## Materials and methods

### Study system

**Fig. 1:**
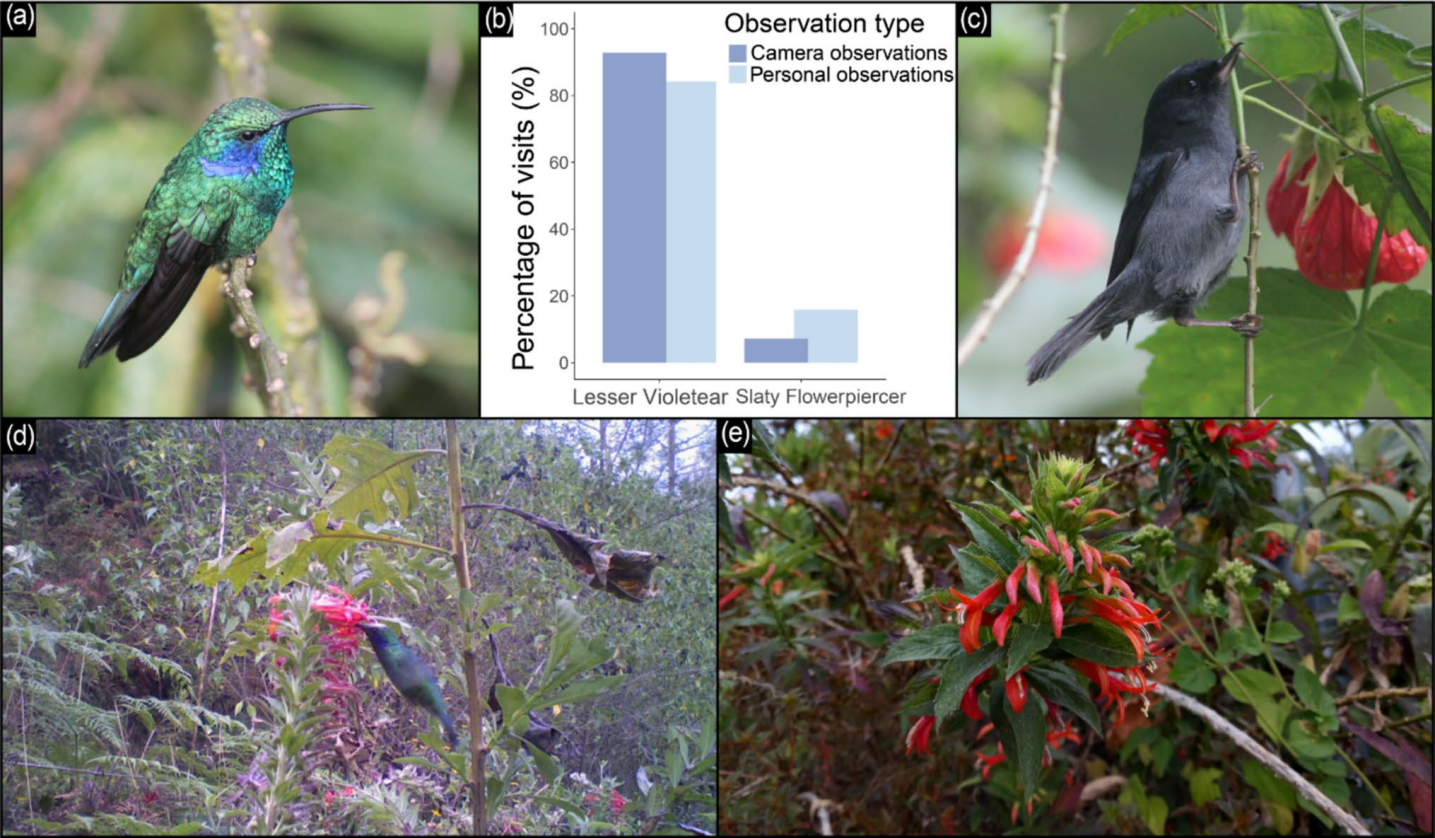
Flower visitors and interaction frequency of (**b** and **e**) *Lobelia laxiflora* (Campanulaceae) in the highlands of the Talamanca mountain range, Costa Rica. Legitimate visitation by (**a** and **d**) the Lesser Violetear (*Colibri cyanotus*) far exceeded that (c) of the Slaty Flowerpiercer (*Diglossa plumbea*). Photo credits: **a** and **c** María A. Maglianesi, **d** *Lobelia*-*Colibri* interaction recorded by a PlotWatcher Pro camera, **e** Stefan Abrahamczyk

*Lobelia laxiflora* is a perennial herb to sub-shrub, up to 3 m tall, forming terminal, flower-rich racemes (Lammer et al. 2004), which bloom from the bottom to the top. Blooming time is from early February to late March in Costa Rica (own obs.). The orange-red, tubular flowers are up to 4.2 cm long, with dorsally largely free corolla tubes (Fig. 1e; Velázquez and Ornelas 2010). Per inflorescence up to 20 flowers are open at the same time. The flowers are hermaphroditic and last around 5 days, being in the staminate phase for 2 days and then for 3 days in the pistillate phase (Velázquez and Ornelas 2010). *Lobelia laxiflora* is distributed in the mountain ranges (250–3450 m) from Arizona to Colombia, but the distribution in the southern part of the range (Costa Rica, Panama, Colombia) is scattered (Lammer et al. 2004).

Our study area is situated in central-southern Costa Rica around the small village of Macho Mora, 17.5 km north of San Isidro de El General in the Talamanca mountain range at 2100–2450 m elevation. Here, *Lobelia laxiflora* grows in a semi-open, cultural landscape on volcanic ash. The population in this area contains several thousand plant individuals and is separated from other populations by a few kilometres direct line. We collected one herbarium specimen of *Lobelia laxiflora*, which is deposited in the herbarium of Stuttgart (STU) in Germany (accession number SMNS-STU-PH-0159775) under research permit R-SINAC-PNI-ACLAP-016–2024 and export permit of biological material CUSBSE-629–2024 (Resolution No. PE-CUSBSE-084–2024).

### Floral traits

We measured morphological flower traits, such as corolla tube length, horizontal and vertical diameter of the opening of the corolla tube and the length of the style including the stigma from three flowers of twelve plant individuals each (in total 36 flowers). For the measurements, we photographed all flowers in a standardized way and calculated the traits digitally using Fiji ImageJ.

To determine the mean number of pollen grains per flower, we collected two flower buds from 12 plant individuals each (total 24 flower buds). We transferred the closed anthers of each bud into a plastic tube and left them to air dry for 2 days with the lid open. Then, we added 200 μL of glycerol to each tube and mixed the suspension for 5 min at 200 Hz using a laboratory mixer mill (Retsch MM 200; Retsch, Haan, Germany). Subsequently, we placed the tubes in an ultrasonic bath (Sonorex Rk 52; Bandelin, Berlin, Germany) for 15 min each and vortexted the mixture before we transferred 20 μL of each sample into a hemocytometer with a Fuchs-Rosenthal counting chamber with 16 squares. Finally, we counted the pollen grains in five randomly chosen squares under a microscope (Axio, Scope.A1, Zeiss) and calculated the total number of pollen grains per flower based on the mean number of pollen grains in the counted squares and the added volume of the suspension.

We used the same flowers as for the pollen count to determine the number of ovules per flower. After collection, we transferred the ovaries into plastic tubes containing ethanol. In the lab we carefully opened the ovary under a stereo microscope (SteREO Discovery.V8, Zeiss) and counted the number of ovules. After opening the ovaries, we realized that 6 of the 24 samples did not contain ovules because they were consumed by insect larvae, which reduced the number of analysed ovules to 18 coming from ten plant individuals.

### Observations of flower visitors

We combined direct observations with recording from unattended cameras. We conducted 45 periods of personal observation of 20 min each (15 h in total) at eight different days between 11 th of February to 20 th of March 2024, coinciding with the flowering season of *L. laxiflora*. We concentrated our observation to the morning hours between 6:00 am and 11:00 am, a time when birds are very active. During the observations, we waited quietly around eight meters away from a clump of *Lobelia* plants of up to ten square meters cover. The number of observed flowers ranged from 25 to 500 (mean 142.5 ± 145.7), depending on the density and size of plants.

We set up five time-lapse cameras that record an image every second (PlotWatcher Pro, Day 6 outdoor), each observing small clumps of plants (1 m^2^ cover) between 5:30 am and 6:00 pm between 13 th of February and 6 th of March 2024 (287.5 hours of observation/camera, 1437.5 h in total). The number of observed flowers ranged from 40 to 50. The images were processed by using DeepMeerkat software (Weinstein 2018) that extracts the images with flower-visiting birds to be manually identified along with other relevant data, such as illegitimate visits, i.e., when the bird made a hole or used one made by another organism in the flower base to obtain the nectar rather than the flower opening.

Additionally, we analysed possible preferences for one flower sex by the two species of flower visitors. For the hummingbirds, we choose 60 random flower visits from the photo material of the cameras, at which the sex of the visited flower was visible. For flowerpiercers, we analysed the distribution of flower sex at all visited flowers with identifiable flower sex.

### Pollinator traits

We collected data on bill length of *Colibri cyanotus* from literature (Maglianesi et al. 2014; Rojas-Rodríguez et al. 2023). For information on bill morphology of *Diglossa plumbea*, we took advantage of unpublished data collected during a project in central Costa Rica in 2015/16. During this project, birds were mist netted and their species identity and sex was noted. Further, total and exposed culmen length was measured.

### Pollination experiments

We conducted two pollination experiments to analyse the reproductive system of *Lobelia laxiflora* and to analyse pollination efficiency of the different pollinator species. For both experiments, we used medium-sized plant individuals with three to five flowering stems. The first (reproductive) experiment had three treatments. For each treatment, we used three to five flowers from 12 plant individuals each. The flowers were individually marked and covered with nets (2 mm mesh width) before the start of the pistellate phase: In the first (autogamous selfing) treatment, we just covered the flowers (45 in total) and did not manipulate them further. In the second (manual self-pollination) treatment, we manually pollinated the flowers (46 in total) with pollen of the same plant individual. In the third (manual cross-pollination) treatment, we manually pollinated the flowers (48 in total) with pollen of another plant individual. In treatments two and three, we applied anthers from three different flowers for the pollination of each flower. We used tweezers to transfer the pollen by dabbing the anthers on the stigmas. After manual pollination, we covered the flowers again until the corolla fall off.

The second (pollination efficiency) experiment had two treatments. In the first one (open pollination treatment), we marked 15 to 19 flowers from 12 plant individuals each (198 flowers in total) but neither covered them nor manipulated them further. In the second (single visit treatment), we marked and covered 15 to 22 flowers (211 flowers in total) per plant individual of the same twelve plant individuals used in treatment 1. All flowers were at the end of the staminate phase. After the beginning of the pistillate phase, we opened the cover again and observed pollinator interactions. After single visits of hummingbirds or flowerpiercers, we covered the flowers again and noted the pollinator species. The total observation time for this part of the experiment were 12 h.

For both experiments, we counted fruit set after 3 weeks, shortly before the fruits became ripe. Further, we collected the nearly ripe fruits and stored them in paper bags to later count the number of developed seeds under a binocular.

### Statistics

We conducted a GLMM with a negative binomial family to explain the relationship between the number of flowers visited and the number of flowers available during personal field observations. Observer was used as a random effect, and an autoregressive variance–covariance matrix of order one was applied to address temporal correlation. This approach was used to exclude the possibility that the presence of humans influenced hummingbird behaviour. We tested the distribution of flower sex of the flowers visited by hummingbirds vs. flower piercers by a Fisher´s exact test. To test for significant differences in seed set among pollination treatments (open pollination, single visit, manual outcrossing), we conducted a Kruskal–Wallis test followed by a Dunn (posthoc) test since the data did not show homogeneity of variance. All analyses were conducted in R 3.4.3 (R Development Core Team 2018).

## Results

### Floral traits

The corolla tube of *Lobelia laxiflora* has a mean length of 1.86 (SD ± 0.18) cm, a mean vertical diameter of 0.53 (SD ± 0.05) cm and a mean horizontal diameter of 0.5 (SD ± 0.04) cm. The style has a mean length of 3.4 (SD ± 0.27) cm. The stamens form a tube around the pistil with the anthers at the tip of the tube. In the pistillate phase, the stigma grows through the tip of the anther tube. The flowers produce a mean number of 369,800 (SD ± 22,722) pollen grains and 1412 (SD ± 403) ovules.

### Observations of flower visitors

During 33 out of the 45 personal observation slots of 20 min each, we observed flower visiting birds in *Lobelia laxiflora* (Fig. 1b). The birds visited two to 296 flowers per time slot (Median 7.5 ± SD 59.2). The number of visited flowers was significantly related to the number of observed flowers (*β* = 0.01, *z* = 4.91, *p* < 0.001, marginal *R*^2^ = 0.36). Most visits (84.2 %) were conducted by the Lesser Violetear (*Colibri cyanotus*; Fig. 1a and d) and the rest (15.8 %) by the Slaty Flowerpiercer (*Diglossa plumbea*; Fig. 1c). Both species visited the flowers without piercing them. *Colibri cyanotus* hovered in front of the flowers and always inserted its bill into the flower opening more or less in the same angle. In contrast, *Diglossa plumbea* used a perch below or beside the flowers depending on the local vegetation structure. Flowerpiercers often visited the lowest open flower, which were mostly in the female phase. Depending on its position to the flower, the bird pushed its bill deeply into the corolla tube, mostly from the side moving the style upwards and out of the dorsally open corolla tube. With this technique, the flowerpiercers reduce the corolla length and to get closer to the nectar without piercing the flowers. As a consequence, different parts of the bird´s body come in contact with the reproductive organs of the flower and sometimes no contacts were observed.

During 1437.5 h of observations by camera, we observed 347 visits of birds. Most visits (92.8 %) were conducted by the Lesser Violetear (*Colibri cyanotus*) and the rest (7.2 %) by the Slaty Flowerpiercer (*Diglossa plumbea*). Again, all visits were non-destructively. Hummingbirds did not show a preference for flower sex (31 visited flowers in male phase vs. 29 flowers in female phase). Flowerpiercers visited 12 female and only one male flower. The distribution of flower sex of the flowers visited by hummingbirds vs. flower piercers differed significantly (Fisher´s exact test, odd ratio = 12.48, *p* = 0.0045).

### Pollinator traits

*Diglossa plumbea* has a mean total culmen length of 11.4 mm (SD ± 1.2 mm) and an exposed culmen length of 7.8 mm (SD ± 0.7 mm). Mean total and exposed culmen length is slightly higher in males (*N* = 35; total: 11.5 mm SD ± 1.3; exposed: 7.9 mm SD ± 0.8) than in females (*N* = 31; total: 11.4 mm SD ± 1.0; exposed: 7.7 mm SD ± 0.5).

### Pollination experiments

The autogamous selfing as well as the manual self-pollination treatment did not lead to any fruit set whereas the manual outcrossing treatment led to a fruit set of 81.3 %. Per developed fruit we counted a mean of 91.7 seeds (SD ± 59.1) after manual outcrossing. The open pollination treatment led to a fruit set of 98 % and the developed fruits had a mean of 988.5 well-developed seeds (SD ± 381.1). Seed set of open pollination was finally calculated from only 44 flowers from 11 plant individuals since 4 capsules got destroyed during transport.

**Fig. 2:**
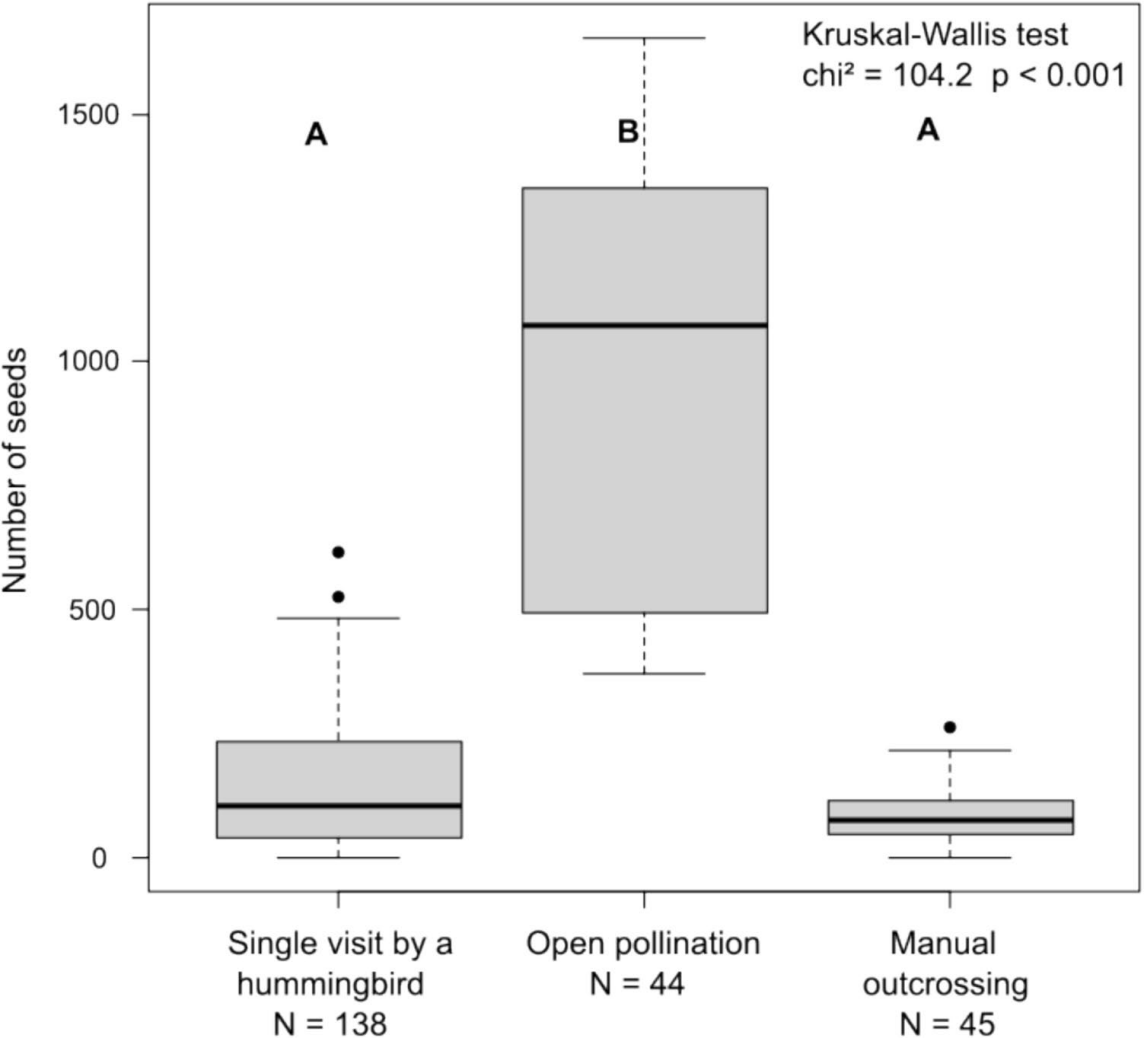
Seed set of *Lobelia laxiflora* after three treatments of pollination experiments in the Talamanca mountain range, Costa Rica for a total sample size (N) of 227 fruits. Different capital letters indicate statistically significant differences after Dunn (post hoc) test for pairwise comparisons. Note that neither the autogamous selfing, nor the manual self-pollination treatment or single visits by flower piercers led to any fruit set

In the single visit experiment, we observed that 164 of the 211 flowers were visited by *Colibri cyanotus* involving all 12 plant individuals. *Diglossa plumbea* visited only 18 flowers of four plant individuals. The visits of *Colibri cyanotus* led to a fruit set of 84.1 % with a mean of 148.7 seeds per fruit (SD ± 138.2). After *Diglossa plumbea* visits, we did not observe any fruit development. Seed set after open pollination was significantly higher than seed sets after single visits of hummingbirds or manual outcrossing (Fig. 2), whereas seed set after manual outcrossing was not significantly higher than after single flower visits of hummingbirds.

## Discussion

In this study, we analyse the reproductive biology of *Lobelia laxiflora* in central-southern Costa Rica and evaluate pollinator efficiency. *Lobelia laxiflora* is a perennial, obligately outcrossing herb in our study region and we never detected any selfing ability in the pollination experiments. The orange-red, long-tubed flowers of *Lobelia laxiflora* produce large amounts of nectar (Velazquez and Ornelas 2010), which attracts nectar-feeding birds. In the studied population, the flowers of *L. laxiflora* are visited exclusively by two bird species: *Colibri cyanotus* conducted the vast majority of visits while *Diglossa plumbea* was a secondary visitor*.* Both bird species visit the flowers without damaging any flower organ. Morphologically, the flower size and morphology of *Lobelia laxiflora* (e.g. corolla length 1.86 cm, horizontal corolla width 0.5 cm) nicely fits the bill morphology of *Colibri cyanotus* (bill length: 2.1 cm; Maglianesi et al. 2014; Rojas-Rodríguez et al. 2023), its main flower visitor. We commonly observed that the stamens protruding from the corolla placed large amounts of pollen on the heads of *Colibri cyanotus*. This morphological fit between interacting partners has already been observed in numerous studies (summarized e.g. in Abrahamczyk and Kessler 2015 or Leimberger et al. 2022) and is interpreted as diffuse co-evolution. In contrast, *Diglossa plumbea* has a much shorter bill (mean 0.78 cm) and is not able to hover in front of the flowers but needs a perch beside or below the flowers. It reached the nectar mostly by entering the bill deeply into the dorsally open corolla tube. Therefore, pollen was not be placed exactly on the heads of *Diglossa plumbea* but depending on the bird´s position on different parts of the bird´s body or the reproductive organs did not come in contact with the bird at all. Further, *Colibri cyanotus* visited both flower sexes equally whereas *Diglossa plumbea* preferred the lowest, open flowers. Due to the opening pattern of the inflorescences of *Lobelia laxiflora*, these were mostly flowers in the female phase. Thus, due to their morphological non-match and strong preference of female flowers flowerpiercers did not act as pollinators for *Lobelia laxiflora* (0 % fruit set after single flower visits). These results underline that morphological trait matching between plants and birds as well as the bird´s behaviour play an important role for the plant´s reproductive success.

*Colibri cyanotus* is a very frequent pollinator, visiting 30 % (mean: 36.7 out of 123.7 flowers) of the observed flowers during direct observations of flower visitors. Single visits of *Colibri cyanotus* led to a fruit set of 84.1 % and a seed set in the developed fruits of 15.1 % in relation to the mean seed set of the open pollination experiment. Further, in the open pollination experiment, 70% of the available ovules developed to seeds. These results indicate that multiple flower visits of hummingbirds were necessary to reach full seed set and that multiple visits per flower commonly occurred. In conclusion, our results document a relatively high pollination efficiency of *Colibri cyanotus* compared to a study on pollination efficiency of morphologically different hummingbird species pollinating *Vriesea rodigasiana* (Bromeliaceae; Rocca and Sazima 2013).

We find several reasons for this high pollination efficiency of *Colibri cyanotus*: 1. *Lobelia laxiflora* was very abundant in our study area and each plant produced one to several inflorescences with up to 20 open flowers per inflorescence. Hummingbirds often moved between plants conducting outcrossing. 2. High morphological fit of flower organs of *Lobelia laxiflora* and the bill length of *Colibri cyanotus* allowed exact pollen placement*.* 3. We never observed visits of *Colibri cyanotus* at co-flowering plant species, such as *Bomarea costaricensis* (Alstroemeriaceae) or *Lamourouxia lanceolata* (Orobanchaceae) leading potentially to interspecific pollen deposition on the stigmas of *Lobelia laxiflora*. 4. During our extensive observation time (> 1450 h), we never observed visits of other hummingbird species at the flowers of *Lobelia laxiflora*, even though other species with fitting bill morphology were present in our study area (e.g. *Eugenes spectabilis* or *Lampornis castaneoventris*). A variety of partly closely related hummingbird species interact with other populations of *Lobelia laxiflora* in Mexico (Lara and Ornelas 2002; Arizmendi et al. 2021; López-Flores et al. 2024). Here, *Lobelia laxiflora* is visited by up to four hummingbird species that vary in their bill morphology and visitation frequency but no information on the contribution of the different hummingbird species to seed set are presented. However, visits of a diverse assemblage of hummingbird species may lead to a larger number of interspecific pollen grains on the stigmas of *Lobelia laxiflora* and can result into stigma clogging*.*

## Conclusions

Our study documents a rare case of a temporally limited one-to-one dependency of a plant and a hummingbird species on the population level. Extending our work to other plant species and populations with variable numbers of interacting partners will give insights into interactions among visitation frequency and morphological fit and its implications for seed set.

Such an extension is essential to determine the actual role of pollinators beyond interaction frequency and whether morphological trait matching is a mechanism that consistently increases pollination efficiency, with important ecological and evolutionary implications in plant-pollinator networks.

## Data Availability

All code and relevant data files are available on Zenodo. The link will be provided later.
